# Should systematic reviews and meta-analyses include data from preprints?

**DOI:** 10.47626/2237-6089-2021-0324

**Published:** 2023-03-07

**Authors:** Elisa Brietzke, Fabiano A. Gomes, Fernando Gerchman, Rafael C. R. Freire

**Affiliations:** 1 Department of Psychiatry Queen’s University School of Medicine Kingston ON Canada Department of Psychiatry, Queen’s University School of Medicine, Kingston, ON, Canada.; 2 Kingston General Hospital Kingston Health Science Centre Kingston ON Canada Kingston General Hospital, Kingston Health Science Centre, Kingston, ON, Canada.; 3 Centre for Neuroscience (CNS) Queen’s University Kingston ON Canada Centre for Neuroscience (CNS), Queen’s University, Kingston, ON, Canada.; 4 Department of Psychiatry and Behavioural Neurosciences McMaster University Hamilton ON Canada Department of Psychiatry and Behavioural Neurosciences, McMaster University, Hamilton, ON, Canada.; 5 Divisão de Endocrinologia e Metabolismo Hospital de Clínicas de Porto Alegre Porto Alegre RS Brazil Divisão de Endocrinologia e Metabolismo, Hospital de Clínicas de Porto Alegre (HCPA), Porto Alegre, RS, Brazil.; 6 Programa de Pós-graduação em Ciências Médicas Faculdade de Medicina Universidade Federal do Rio Grande do Sul Porto Alegre RS Brazil Programa de Pós-graduação em Ciências Médicas, Faculdade de Medicina, Universidade Federal do Rio Grande do Sul (UFRGS), Porto Alegre, RS, Brazil.

As attentive observers of the rapidly developing literature on the effect of the Covid-19 pandemic in mental health, we have been witnessing an interesting conundrum: should systematic reviews and meta-analyses include data from preprints in their results?^1^

Publication of manuscripts in a traditional peer-reviewed journal can take a long time, while preprints are readily available online. Preprint articles were already becoming more common, mainly due to the need to protect intellectual property, but preprint platforms gained a remarkable impulse since the arrival of the Covid-19 pandemic.^2^ Today, there is a pressing need to make data on Covid-19 available as quickly as possible and the scientific community has been willing to be more flexible in terms of what would be the ideal process to consider results as both scientifically valid and clinically useful.

Searching for unpublished data is common when conducting systematic reviews and meta-analyses. The so called ‘grey literature’ has been a source of data for these types of studies and it is common to read in the methods of systematic reviews and meta-analyses that authors have also searched for conference abstracts or even personal communications between researchers. It is thought that this type of search could help to reduce publication bias, providing readers a more balanced view of the knowledge on the topic.^3^ In addition, the fact that a study has not been peer-reviewed does not necessarily mean it lacks quality or clinical applicability.

In a recent study, Carneiro et al. contrasted the quality of data reporting in independent samples of preprints with published studies.^4^ They also made pairwise comparisons of preprint versions with their published versions. They found that, on average, the quality of reporting is slightly better for peer-reviewed journal articles compared to preprint articles. Compared to peer-reviewed articles, preprint articles were less likely to report conflicts of interest, funding sources, and unit-level (most commonly individual people) data. These data are not in accordance with other studies which did not find significant differences between the two versions.^5-7^ Conflicts of interest are a very relevant variable which is commonly used in sensitivity analyses in meta-analyses of interventional studies.^8^ It should be noted that preprint repositories have recently changed their policy for reporting conflicts of interest, with medRxiv requiring disclosure of conflicts of interest, while bioRxiv was not requiring it at the time the study by Carneiro et al. was conducted.^4^

Preprint articles have the unquestionable advantage of quick availability online. While an article may take several months or even more than a year to be published in a journal, publication on a preprint server takes less than a week. Currently, many journals are applying fast-tracked processes to articles related to COVID-19, but the peer-review process still requires time for qualified referees.^9^

To add an additional layer of complexity, Schmucker et al. performed a methodological research study of systematic reviews and meta-analyses in medicine.^10^ Their aim was to investigate if including unpublished data would have an impact on results. They found that, although there were some cases that not including unpublished data led to an overestimation of the results, this only affected a minority of reviews. However, when researchers propose systematic reviews and meta-analyses in which a significant volume of information will come from unpublished material, this generates new sources of biases. For example, most preprint datasets do not provide a search strategy that is equivalent to major databases, such as PubMed. So, it becomes impossible to guarantee the reproducibility of the retrieval, because how many of the studies would or would not be found is subject to significant variation.

This is only one of the arguments against inclusion of preprints in systematic reviews and meta-analyses. Other criticisms are:

1. The possibility that the results change further and the findings of the review and meta-analyses must also be modified;
2. Some preprints will never pass the minimum quality criteria for publication and in theory should be excluded from a meta-analysis;
3. Possibility of conflicting information between the preprints and the final form of the published article (e.g. authorship, additional analysis, discussion of the results). Notwithstanding, these challenges should not lead us to ignore that results on a research question were generated, even when they are made public only in a preprint.

Future approaches to this topic could include the obligation to disclose conflicts of interests, funding, and unit-level data in preprints, a better description of the search strategy in the preprint datasets, improvement of the advanced search tools for the preprint datasets, standardized analysis of the quality of the gray literature and preprints, inclusion of additional exploratory analysis of peer-reviewed and not peer-reviewed material, and, finally, a balanced discussion of the impact of inclusion of these two types of literature on future systematic reviews and meta-analyses. It also should be highlighted that, irrespective of the inclusion criteria adopted in systematic reviews and meta-analyses, they should be considered temporary and be updated periodically. The improvement of meta-science methods can be a good collateral effect of the Covid-19 pandemic. It may not only benefit the literature in the field but also other subjects in mental health, accelerating interpretation of evidence and knowledge translation.
